# The higher adherence to a healthy lifestyle score is associated with a decreased risk of type 2 diabetes in Iranian adults

**DOI:** 10.1186/s12902-022-00961-4

**Published:** 2022-02-17

**Authors:** Hossein Farhadnejad, Farshad Teymoori, Golaleh Asghari, Ebrahim Mokhtari, Parvin Mirmiran, Fereidoun Azizi

**Affiliations:** 1 Nutrition and Endocrine Research Center, Research Institute for Endocrine Sciences, Shahid Beheshti University of Medical Sciences, Tehran, Iran; 2 Department of Clinical Nutrition and Dietetics, Faculty of Nutrition Sciences and Food Technology, National Nutrition and Food Technology Research Institute, Shahid Beheshti University of Medical Sciences, Tehran, Iran; 3 Department of Nutrition, School of Public Health, University of Medical Sciences, Tehran, Iran; 4 Endocrine Research Center, Research Institute for Endocrine Sciences, Shahid Beheshti University of Medical Sciences, Tehran, Iran

**Keywords:** Healthy lifestyle score, Dietary pattern, Obesity, Physical activity, Smoking, Type 2 diabetes, Adult

## Abstract

**Background:**

The combined roles of lifestyle factors in the risk of type 2 diabetes (T2D) are not fully investigated. In the present study, we aimed to assess the relationship between a healthy lifestyle score (HLS) and the risk of T2D in Tehranian adults.

**Methods:**

A total of 3859 individuals without T2D were recruited from participants of the Tehran Lipid and Glucose Study (2009–2011) who were followed up for a mean period of 6 years. A food frequency questionnaire was used to collect individuals' dietary intakes at baseline. HLS scores was calculated based on three pre-defined methods with focusing on 4 lifestyle factors including, no current smoking, no obesity, high physical activity, and greater adherence to the healthy diet[determined using the alternate healthy eating index-2010(AHEI-2010), modified French Programme National Nutrition Santé-Guideline Score(mPNNS-GS), and healthy diet pattern score(HDP)].

**Results:**

Mean ± SD age of participants(44.4% men) was 41.1 ± 12.3 years. After 6-year follow-up of study, 295(7.6%) new cases of T2D were reported. Based on the age and sex-adjusted model, an inverse association was observed between the higher score of HLS-AHEI-2010 (OR = 0.24;95%CI:0.10–0.60), HLS-mPNNS-GS (OR = 0.28;95%CI:0.15–0.50), and HLS-HDP (OR = 0.39;95%CI:0.24–0.64) and the risk of T2D (*P* for trend < 0.05). Also, the fully-adjusted model showed that after controlling the effects of various confounders, this invers association between the higher score of HLS-AHEI-2010 (OR = 0.25;95%CI:0.10–0.61, P for trend:0.001), HLS-mPNNS-GS (OR = 0.29;95%CI:0.15–0.55,P for trend:0.001), and HLS-HDP (OR = 0.36;95%CI:0.22–0.61,*P* for trend < 0.001) and risk of T2D was remained significant.

**Conclusion:**

The results of this study suggested that higher score of HLS, characterized by no smoking, normal body weight, vigorous physical activity, and healthy diet, is related to decreased risk of T2D incidence.

## Background

Type 2 diabetes (T2D) is a major health problem with complex metabolic conditions, which is considered the leading preventable risk factor for the development of cardiovascular disease (CVD) and all-cause mortality worldwide [1, 2]. Indeed, T2D is a complex and polygenic disease characterized by chronic hyperglycemia as a consequence of impaired insulin action and secretion, insulin resistance, unfavorable inflammatory condition, and metabolic susceptibility [3]. In 2017, 8.5% of the global adult population (425 million people) had T2D, and it has been anticipated to be 629 million by 2045 [1]. Recent investigation has reported that the T2D incidence rate in the Iranian population was 36.3 per 1000 person-years, with more than 800,000 new cases per year [4]. Also, it is estimated that in the year 2030, nearly 9.2 million Iranians will be affected by T2D [5]. Lack of proper management of T2D with the increasing duration of this disease threatens people's lives due to various microvascular complications such as nephropathy, retinopathy, and neuropathy, and macrovascular complications in the form of peripheral vascular disease and coronary artery disease and lead to premature death [1, 6].

The interplay of genetic predisposition and metabolic factors are the main drivers of the global epidemic of T2DM [7]. Along with population aging, modifiable factors, including unhealthy dietary pattern, obesity, physical inactivity, and smoking, are recognized as prominent risk factors that can contribute to the development of T2D [1, 2]. In previous reports, lifestyle risk modification has been suggested as a strong strategy in the prevention of chronic diseases such as T2D [8]; so that it has been suggested that variable levels of each modifiable lifestyle factor, including smoking [9], physical activity [10], normal body weight [11], and adherence to a healthy dietary pattern [12] can be associated with the risk of T2D. Also, some studies have recently investigated the combined role of lifestyle factors, including healthy diet pattern, not smoking, avoiding obesity, and regular exercise, as a single variable, called healthy lifestyle score (HLS), with risk of chronic diseases and mortality [13–16]. Based on the results of previous reports, adherence to HLS is related to the promotion of overall health during aging [13], decreasing the risk of coronary disease [14], and reducing mortality in type 2 diabetic subjects [15, 16]. However, to the best of our knowledge, there is no study on the association of combined lifestyle factors with the risk of T2D incidence in the adult population.

Considering convincing evidence on the high incidence of T2D among Iranian adults [17], the possible beneficial combined role of healthy lifestyle factors as an HLS in the prevention of some chronic diseases or their related mortality, and also due to the lack of data on the association between HLS and the risk of T2D, in the present study, we aimed to assess the relationship between HLS and the risk of T2D incident among Iranian adult population.

## Methods

### Study population

The present study was conducted within the framework of the Tehran Lipid and Glucose Study (TLGS), a population-based cohort study that was performed to determine the risk factors for chronic diseases among a representative urban population of Tehran, including 15,005 participants aged ≥ 3 years [18]. The first phase of TLGS (a cross-sectional survey) was initiated in March 1999, and data collection, conducted prospectively at three years intervals, is ongoing; the details of the TLGS have been reported previously [18]. In the fourth survey of the TLGS (2009–2011), of 12,823 participants, 7956 subjects, aged 3–75 years, were randomly selected to be assessed for dietary intakes.

**Fig. 1:**
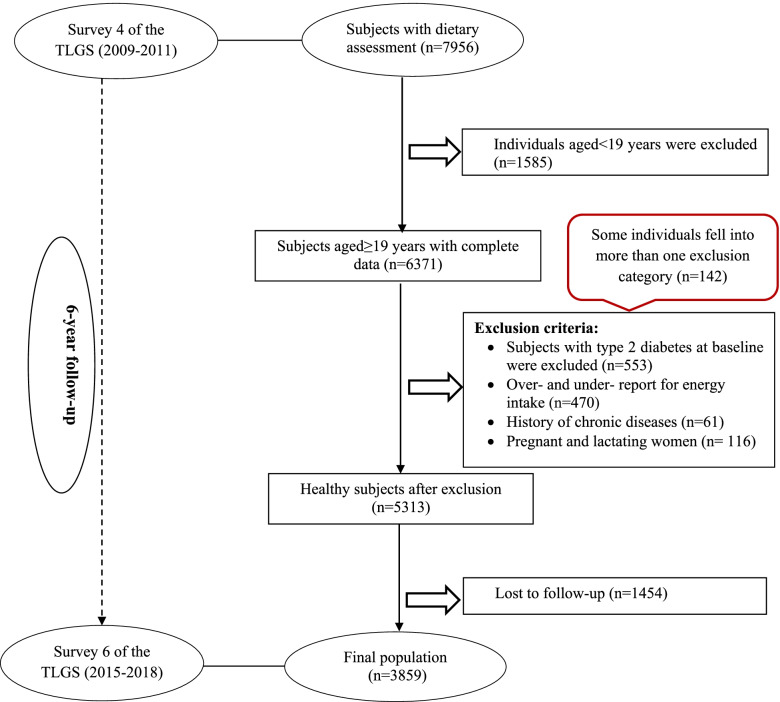
Flow chart of the Tehran Lipid and Glucose Study (TLGS) participants

For the present study, after excluding individuals aged < 19 years (*n* = 1585), 6371 participants, aged ≥ 19 years, with complete data in the fourth phase of the TLGS (baseline examination), were selected. Individuals who under- or over-reported dietary intakes (< 800 kcal/day or > 4200 kcal/day, respectively) or who were on specific diets (*n* = 470), those with prevalent cancer (*n* = 12), cardiovascular diseases (*n* = 49), and pregnant and lactating women (*n* = 116), were excluded. The individuals with baseline T2D were also excluded (*n* = 553); some of whom fell into more than one category (*n* = 142). Next, 5313 healthy individuals (free of T2D) were followed until the sixth examination, for a mean period of 6 years from the baseline phase (fourth survey). Finally, after excluding the participants who left the study (*n* = 1454), final analyses were performed on the data of 3859 adults (Fig. 1).

#### Physical activity assessment

Data on physical activity was collected using a modifiable activity questionnaire (MAQ), which was previously modified and validated among Iranian adults [19]. Individuals were asked to report and identify the frequency and time spent on activities of light, moderate, hard, and very hard intensity over the past 12 months, based on a list of usual activities of daily life; the 'participants' physical activity levels were reported as metabolic equivalent hours per week (MET-h/wk).

#### Demographic, anthropometric, and lifestyle assessment

Details of the TLGS data collection approaches and measurements have been previously reported [18]. The participants were asked to report their information on demographic characteristics, drug use, medical history, and cigarette smoking using a pretest standard questionnaire at baseline of current study. Body weight was measured and recorded to the nearest 100 g using a digital scale while participants wearing minimal clothes and were without shoes. The height of individuals was determined to the nearest 0.5 cm using a stadiometer, without shoes that shoulders were in a normal position. Body mass index (BMI) was considered as a individual's weight in kilograms divided by the square of their height in meters. The systolic blood pressure (SBP) and diastolic blood pressure (DBP) were determined using a standardized mercury sphygmomanometer with an accuracy of two mmHg based on standard protocols which was previously explained [18].

#### Biochemical Measurements

A 10 ml blood sample was taken after 12–14 h of overnight fasting to measure biochemical variables including fasting plasma glucose (FPG), triglycerides (TGs), total cholesterol (TC), and high-density lipoprotein-cholesterol (HDL-C) based on the standard protocol that has been previously descriebed [20]. All blood analyses were performed at the TLGS research laboratory using the Selectra 2 auto-analyzer (Vital Scientific, Spankeren, The Netherlands) to analyze the samples. FPG, TG, TC, and HDL-C were measured using enzymatic colorimetric method and all analyses were done using commercial kits (Pars Azmoon Inc., Tehran, Iran). The Friedewald formula was used to determine low-density lipoprotein-cholesterol (LDL-C) from the serum TC, TG, and HDL-C concentrations.

#### Dietary assessment

Dietary intakes of individuals were collected using a valid and reliable semi-quantitative food frequency at baseline [21]. The trained nutritionist asked participants to designate their consumption frequency for each food item during the previous year daily, weekly, or monthly; portion sizes of consumed foods, reported in household measures, were then converted to grams. Since the Iranian Food Composition Table (FCT) is incomplete and has limited data on the nutrient content of raw foods and beverages, the United States Department of Agriculture (USDA) FCT was used. The Iranian FCT was used for national foods not listed in the USDA FCT.

#### Determination of healthy lifestyle scores

The score of healthy lifestyle factors for participants was determined using three healthy lifestyle indices, including HLS- alternate healthy eating index (AHEI)-2010 [15], HLS- modified French Programme National Nutrition Santé-Guideline Score (mPNNS-GS) [13], and HLS-healthy dietary pattern (HDP) [14]. All three indices consist of four factors, including smoking (yes/no), physical activity (active/ inactive), obesity (yes/no), and diet quality, which in each of these scoring procedures, the quality of the diet has been assessed in different ways.

For computing of HLS-AHEI-2010 [15], individuals were divided into two groups based on each lifestyle factor as follows: non-obese (BMI < 25 kg/m^2^) vs. overweight/obese (BMI ≥ 25 kg/m2); non-current smoking vs. current smoking; physically active (≥ 22.5 MET/h/w) vs. physically low active/sedentary (< 22.5 MET/h/w). A score of 1 was assigned to participants who were normal weight. Also, a score of 1 was considered for subjects who were non-smoked. Furthermore, the physically active participants (≥ 22.5 MET/h/w) received a score of 1. The quality of diet in participants has been assessed based on the AHEI-2010 diet score, which was defined in detail previously [22]. Individuals were divided into quintiles based on the AHEI-2010 score; individuals in the upper two quintiles of the AHEI-2010 score were given a score of 1 (as a higher adherence to a healthy diet). The points for factors were then summed to create the overall healthy lifestyle score, which ranged from 0 (lowest adherence to HLS-AHEI-2010) to 4 (highest adherence to HLS-AHEI-2010).

The HLS-HDP index was also determined according to the strategic goals of the American Heart Association (AHA) using four important lifestyle variables (as binary components), including BMI, physical activity, smoking, diet quality [14]. We assigned a score of 1 to participants who are not obese (BMI < 30 kg/m^2^), are non-current smoking, and are physically active (≥ 66.5 MET/h/w). The quality of dietary pattern in participants was assessed by focusing on high consumption of fruits, vegetables, fish, whole grains, nuts, and dairy products and low consumption of processed meats, unprocessed red meats, refined grains, trans fats, sugar-sweetened beverages, and sodium. The overall healthy dietary pattern score ranged from 0 to 12. Then, a score of 1 was assigned to individuals if they had a healthy dietary pattern (diet quality score ≥ 6). Finally, we summed the points for each of the factors to create the overall healthy lifestyle score, which ranged from 0 (lowest adherence to HLS-HDP) to 4 (highest adherence to HLS-HDP).

In the present study, lifestyle score (named as HLS-mPNNS-GS) was also determined based on the method reported in the Atallah et al. study [13]. Like the methods mentioned above, HLS-mPNNS-GS were calculated based on four main lifestyle factors: no obesity, vigorous exercise, no smoking, and a healthy diet. We assigned a score of 1 for participants if they are normal weight (BMI = 18.5–24.99 kg/m^2^) and non-current smoking. Also, participants were divided into quartiles based on physical activity level; individuals in the upper two quartiles of physical activity level were given a score of 1 (as an active people). The diet quality score in participants was calculated based on the French guideline-based dietary index (mPNNS-GS). We have determined the overall healthy diet score based on 10 food groups, including fruit and vegetables, bread and legumes, whole grains, dairy products, red and processed meat, fish and seafood, added fats, fats with the vegetable origin, sugary food, and Sweetened beverage, salt, and MET score. The diet quality score ranged from 0 to 12.5. We assigned a score of 1 to participants if they had a healthy dietary pattern (diet quality score ≥ 6.5). Finally, we summed the points of the factors to create the overall healthy lifestyle score, which ranged from 0 (lowest adherence to HLS- mPNNS-GS) to 4 (highest adherence to HLS- mPNNS-GS).

Since alcoholic drinks intakes such as wine and liquor are unusual among the Iranian people due to religious considerations and were not reported in the TLGS study, we did not include them in computing the three indices mentioned above. As we did not have any food items as low-energy beverages and cream soup in our FFQ, we excluded them from the calculation.

#### Definitions

Diabetes was defined based on the criteria of the American Diabetes Association (ADA) as FPG ≥ 126 mg/dl or 2-h post 75 g glucose load ≥ 200 mg/dl or taking oral hypoglycemic medication [23].

#### Statistical analysis

All analyses of our study were done using the Statistical Package for Social Sciences (Version 20.0; SPSS, Chicago, IL). Kolmogorov–Smirnov test was used to check the variables' normality. Baseline characteristics of the individuals were presented as the mean ± SD or median (25–75 interquartile) for continuous variables and percentages for categorical variables. We used Chi-square and independent two-sample t-tests to compare qualitative and quantitative variables, respectively, between individuals with and without T2D. Individuals were also classified according to quartiles of HLS (HLS-AHEI-2010, HLS-mPNNS-GS, and HLS-HDS) cut-off points; Chi-square test and linear regression analyses were used to test the trends of categorical and continuous variables across quartiles of HLS, respectively. Multivariable logistic regression models were used to predict the risk of 6-year incident T2D (as the dependent variable) across quartiles of HLS-AHEI-2010, HLS-mPNNS-GS, and HLS-HDS (as independent variables). The first quartile of HLS was considered as the reference group and odds ratios (ORs) and 95% confidence intervals (CIs) were reported for two logistic regression models. Potential confounders, including age, gender, energy intake, employment status, education, and marital status, were adjusted in multivariable model. *P*-values < 0.05 were considered to be statistically significant.

## Results

The mean age and BMI in all study population were 41.0 ± 12.3 years and 27.2 ± 4.6 kg/m^2^, respectively. Also, the percentage of men in our study was 44.4%. We identified 295 (7.6%) new cases of T2D after an average of 6 years of follow-up. The characteristics of the T2D patients and non-T2D subjects are expressed in Table 1. Indivduals with T2D had significantly lower academic education and HDL-C, and had higher mean of age, BMI, FPG, TGs, LDL-C, SBP, and DBP in comparison to non-T2D subjects (*P* < 0.001). The findings of Table 1 showed that the % of employed individuals, smoking, men, and the mean of physical activity, energy inakes, and macronutrients did not differ significantly between the T2D and non-T2D groups.

Participants' characteristics are also presented across the quartiles of HLS-AHEI-2010, HLS-mPNNS-GS, and HLS-HDP in Tables 2, 3, and 4, respectively. Based on the results of Table 2, participants in the highest quartile of the HLS-AHEI-2010 score significantly to be more female, younger, non-smoked, had higher levels of physical activity, academic education, and HDL-C, and had a lower level of BMI, TGs, LDL-C, SBP, DBP, and lower % incidence of T2D (after 6-years of follow-up) compared to those in the lowest quartile of HLS-AHEI-2010 (*P* < 0.05). Also, the intakes of vegetables, whole grains, fruits, and nuts and legumes significantly increased across quartile of the HLS-AHEI-2010 (*P* < 0.05), whereas dietary intakes of red and processed meat, sweetened beverages, trans fatty acid, and sodium were significantly decreased across HLS-AHEI-2010 score quartile (*P* < 0.05).

**Table 1:** Baseline characteristics of participants according to the development of the type 2 diabetes

	**Type 2 diabetes** **(*n* = 295)**	**Non-Type 2 diabetes** **(*n* = 3564)**	***P*-value**
**Baseline demographic and biochemical data**			
Age (year)	47.4 ± 11.5	40.6 ± 12.3	< 0.001
Male, %	43.7	44.5	0.423
Body mass index (kg/m^2^)	30.8 ± 5.3	27.0 ± 4.4	< 0.001
Physical Activity (MET/h/w)	65.1 (35.7–93.8)	67.5 (35.7–99.1)	0.660
Academic education (graduated),%	20.5	29.9	< 0.001
Employed, %	84.1	84.7	0.419
Smoking, %	11.9	10.8	0.310
Fasting plasma glucose (mg/dl)	102.0 ± 10.3	92.2 ± 7.9	< 0.001
Triglycerides (mg/dl)	169 (126–230)	113 (80–162)	< 0.001
Low density lipoprotein-Cholesterol (mg/dl)	117.7 ± 32.6	111.6 ± 32.5	0.003
High density lipoprotein-Cholesterol (mg/dl)	44.4 ± 10.5	47.9 ± 11.6	< 0.001
Systolic blood pressure (mmHg)	123.4 ± 18.2	111.9 ± 15.1	< 0.001
Diastolic blood pressure (mmHg)	80.9 ± 12.4	74.9 ± 10.7	< 0.001
**Dietary intakes**			
Energy (kcal/day)	2359 ± 701	2399 ± 709	0.351
Carbohydrate (g/day)	348 ± 113	354 ± 119	0.394
Protein (g/day)	87.3 ± 28.1	89.2 ± 48.8	0.508
Fat (g/day)	77.5 ± 28.1	80.3 ± 48.2	0.315

Data are presented as mean ± standard deviation for normally distributed variables and median (25–75 interquartile range) for skewed variables, and percent for categorical variables.

**Table 2:** General characteristics and HLS-AHEI-2010 components of participants according to quartiles of HLS-AHEI-2010

**Variable**	**Quartiles of HLS-AHEI-2010**				*P* for trend
	Q1 (*n* = 390)	Q2 (*n* = 1642)	Q3 (*n* = 1501)	Q4 (*n* = 326)	
**Mean score**	1.00 ± 0.00	2.00 ± 0.00	3.00 ± 0.00	4.00 ± 0.00	
Age (year)	43.1 ± 11.9	41.4 ± 11.8	40.7 ± 12.7	39.1 ± 13.2	< 0.001
Male, %	53.5	43.2	42.6	48.5	0.001
Academic education (graduated),%	27.6	26.5	30.8	37.0	0.001
Employed, %	81.5	86.3	84.6	80.1	0.352
Fasting plasma glucose (mg/dl)	94.5 ± 9.2	93.9 ± 8.6	92.2 ± 8.1	90.3 ± 7.8	< 0.001
Triglycerides (mg/dl)	140.0 (100.0 – 195.0)	125.0 (89.0 –175.0)	107.0 (77.0 –158.7)	97.0 (70.0 – 137.0)	< 0.001
Low density lipoprotein-Cholesterol (mg/dl)	116.0 ± 34.4	112.7 ± 30.8	111.0 ± 33.1	108.2 ± 34.3	0.005
High density lipoprotein-Cholesterol (mg/dl)	45.1 ± 10.8	47.0 ± 11.3	48.4 ± 11.6	50.9 ± 11.3	< 0.001
Systolic blood pressure (mmHg)	115.5 ± 16.1	113.8 ± 15.2	111.8 ± 16.1	107.8 ± 12.7	< 0.001
Diastolic blood pressure (mmHg)	76.5 ± 10.8	76.1 ± 11.1	74.8 ± 10.9	72.1 ± 9.4	< 0.001
Diabetes incidence (%)^a^	8.0	9.4	6.9	1.8	< 0.001
**Body mass index (kg/m**^**2**^**)**	29.3 ± 3.4	28.4 ± 4.2	26.5 ± 4.7	22.6 ± 1.9	< 0.001
**Smoking (%)**	50.4	11.0	2.8	0.0	< 0.001
**Physical activity (MET/h/w)**	17.8 (8.9 – 89.3)	66.9 (33.1 – 96.5)	71.8 (46.0 – 102.0)	75.8 (45.3 – 105.7)	< 0.001
**Diet quality score (based on AHEI-2010 score)**	55.0 ± 5.7	56.8 ± 6.6	62.3 ± 7.8	67.0 ± 4.0	< 0.001
**AHEI-2010 components**					
Fruit (serving/day)	2.36 (1.28 – 4.03)	2.47 (1.44 – 4.37)	3.30 (1.87 – 5.24)	3.90 (2.46 – 5.61)	< 0.001
Vegetables (serving/day)	2.37 (1.62 – 3.33)	2.76 (1.81 – 3.93)	3.28 (2.08 – 4.61)	3.51 (2.63 – 5.01)	< 0.001
Nut and legumes (serving/day)	0.46 (0.31 – 0.67)	0.51 (0.32 – 0.78)	0.70 (0.41 – 1.05)	0.90 (0.60 – 1.25)	< 0.001
Whole grain (serving/day)	3.31 (1.49 – 5.84)	3.31 (1.66 – 5.93)	3.71 (2.10 – 5.92)	4.05 (2.61 – 6.57)	< 0.001
Red and processed meat (serving/day)	0.73 (0.42 – 1.21)	0.71 (0.43 – 1.16)	0.56 (0.35 – 0.88)	0.51 (0.33 – 0.75)	< 0.001
Sweetened beverage (serving/day)	0.15 (0.05 – 0.39)	0.14 (0.05 – 0.36)	0.12 (0.04 – 0.26)	0.10 (0.03 – 0.21)	< 0.001
Trans fatty acid (g/day)	2.75 (1.21 – 5.31)	2.31 (1.25 – 4.57)	2.31 (1.19 – 4.60)	2.12 (1.07 – 4.58)	0.025
Long chain n-3 poly unsaturated fatty acid (g/ week)	0.42 (0.21 – 0.91)	0.47 (0.22 – 1.19)	0.51 (0.25 – 1.22)	0.53 (0.27 – 1.42)	0.513
Sodium (mg/day)	3555 (2845 – 4384)	3473 (2698 – 4379)	3290 (2616 – 4205)	3266 (2456 – 4162)	0.002

Data are presented as mean ± standard deviation for normally distributed variables and median (25–75 interquartile range) for skewed variables, and percent for categorical variables

^a^Percentage of diabetes incidence across qaurtiles of HLS-AHEI-2010 was determined at the end of follow-up of study

**Table 3:** General characteristics and HLS- mPNNS-GS components of participants according to quartiles of HLS- mPNNS-GS

**Variable**	**Quartiles of HLS-mPNNS-GS**				*P* for trend
	Q1 (*n* = 519)	Q2 (*n* = 1044)	Q3 (*n* = 1757)	Q4 (*n* = 539)	
**Mean score**	1.00 ± 0.00	2.00 ± 0.00	3.00 ± 0.00	4.00 ± 0.00	
Age (year)	44.2 ± 13.2	41.5 ± 12.5	41.3 ± 11.6	36.8 ± 12.3	< 0.001
Male, %	68.9	60.1	51.5	47.5	< 0.001
Academic education (graduated),%	23.1	27.5	29.3	37.8	< 0.001
Employed, %	76.2	82.4	87.9	86.2	< 0.001
Fasting plasma glucose (mg/dl)	93.8 ± 9.1	93.3 ± 8.9	93.2 ± 8.3	90.6 ± 7.1	< 0.001
Triglycerides (mg/dl)	130.0 (92.0 – 185.0)	121.0 (84.0 – 173.0)	120.0 (87.0 – 171.0)	90.0 (66.0 – 128.0)	< 0.001
Low density lipoprotein-Cholesterol (mg/dl)	115.8 ± 33.1	111.9 ± 33.4	113.4 ± 32.0	104.1 ± 30.4	< 0.001
High density lipoprotein-Cholesterol (mg/dl)	47.8 ± 12.4	47.5 ± 11.8	47.0 ± 11.0	49.9 ± 11.2	0.064
Systolic blood pressure (mmHg)	115.2 ± 17.2	112.7 ± 15.5	113.7 ± 15.3	107.2 ± 13.8	< 0.001
Diastolic blood pressure (mmHg)	76.4 ± 11.5	75.1 ± 11.0	76.2 ± 10.9	71.8 ± 9.4	< 0.001
Diabetes incidence (%)^a^	9.5	9.2	7.9	2.2	< 0.001
**Current smoker (%)**	19.0	22.2	5.1	0.0	< 0.001
**Physical activity (MET/h/w)**	26.0 (12.9 – 37.6)	38.7 (19.9 – 71.4)	82.3 (61.5 – 112.6)	89.3 (67.0 – 128.0)	< 0.001
**Body mass index (kg/m**^**2**^**)**	29.3 ± 4.5	27.5 ± 4.5	27.9 ± 4.4	22.7 ± 1.6	< 0.001
**Diet quality score (based on mPNNS-GS)**	6.05 ± 0.69	7.01 ± 1.08	7.96 ± 0.88	8.08 ± 0.80	< 0.001
**mPNNS-GS components**					
Fruit and vegetables (serving/day)	2.00 (2.00 – 3.99)	3.09 (2.00 – 5.31)	3.35 (2.00 – 5.41)	3.40 (2.00 – 5.44)	0.001
Bread and legumes (serving/day)	9.75 (6.87 – 13.34)	9.75 (6.54 – 12.83)	9.93 (6.79 – 13.00)	10.23 (7.82 – 13.90)	0.011
Whole grains (serving/day)	2.85 (1.23 – 5.30)	3.39 (1.77 – 5.81)	3.75 (2.06 – 6.09)	3.66 (2.08 – 6.50)	0.001
Dairy products (serving/day)	1.54 (0.89 – 2.37)	1.66 (1.13 – 2.44)	1.68 (1.11 – 2.48)	1.70 (1.14 – 2.50)	0.884
Red meat (serving/day)	0.64 (0.39 – 0.1.04)	0.63 (0.39 – 1.03)	0.63 (0.39 – 0.96)	0.58 (0.33 – 0.94)	0.003
Sea foods (serving/day)	0.16 (0.09 – 0.25)	0.21 (0.11 – 0.47)	0.24 (0.13 – 0.49)	0.26 (0.14 – 0.52)	< 0.001
Fats (serving/day)	3.62 (1.72 – 5.88)	3.62 (1.91 – 6.29)	3.54 (1.88 – 6.20)	3.92 (2.04 – 6.38)	0.055
Fats of vegetables origin (serving/day)	2.40 (1.24 – 4.58)	2.40 (1.30 – 4.88)	2.45 (1.37 – 5.05)	2.55 (1.30 – 5.42)	0.005
Sweetened foods (serving/day)	1.28 (0.63 – 2.11)	1.40 (0.77 – 2.27)	1.46 (0.80 – 2.32)	1.57 (0.84 – 2.30)	0.098
Sodium (mg/day)	3431 (2668 – 4312)	3431 (2657 – 4315)	3395 (2681 – 4281)	3394 (2732 – 4244)	0.385

Data are presented as mean ± standard deviation for normally distributed variables and median (25–75 interquartile range) for skewed variables, and percent for categorical variables

^a^Percentage of diabetes incidence across quartiles of HLS- mPNNS-GS was determined at the end of follow-up of study

**Table 4:** General characteristics and HLS-HDP components of participants according to quartiles of HLS- HDP

**Variables**	**Quartiles of HLS- HDP**				*P* for trend
	Q1 (*n* = 348)	Q2 (*n* = 1396)	Q3 (*n* = 1578)	Q4 (*n* = 537)	
**Mean score**	1.00 ± 0.00	2.00 ± 0.00	3.00 ± 0.00	4.00 ± 0.00	
Age (year)	44.3 ± 11.7	40.9 ± 12.4	40.7 ± 12.3	40.7 ± 12.0	< 0.001
Male, %	57.3	62.8	53.0	43.4	< 0.001
Academic education (graduated),%	19.4	26.5	31.6	35.3	< 0.001
Employed, %	80.9	82.8	86.1	87.3	0.006
Fasting plasma glucose (mg/dl)	95.2 ± 9.2	93.0 ± 8.7	92.7 ± 8.3	92.1 ± 7.8	< 0.001
Triglycerides (mg/dl)	133.0 (98.0 – 185.0)	118.50 (82.0 – 169.7)	113.0 (80.7 – 165.0)	113.0 (78.0 – 160.0)	0.002
Low density lipoprotein-Cholesterol (mg/dl)	115.0 ± 34.1	112.5 ± 32.3	111.1 ± 32.6	111.7 ± 31.5	0.099
High density lipoprotein-Cholesterol (mg/dl)	45.3 ± 10.7	47.8 ± 11.3	48.0 ± 11.7	48.7 ± 11.9	0.019
Systolic blood pressure (mmHg)	116.7 ± 15.2	112.7 ± 16.1	112.1 ± 15.6	112.1 ± 14.5	< 0.001
Diastolic blood pressure (mmHg)	77.7 ± 10.7	75.1 ± 11.2	75.0 ± 10.8	75.2 ± 10.30	0.011
Diabetes incidence (%)^a^	13.8	9.0	5.9	5.2	< 0.001
**Current smoker (%)**	36.0	14.8	5.6	0.0	< 0.001
**Physical activity (MET/h/w)**	39.6 (22.9 – 56.1)	47.3 (23.8 – 71.4)	78.1 (47.0 – 107.1)	98.2 (81.1 – 129.1)	< 0.001
**Body mass index (kg/m**^**2**^**)**	31.8 ± 7.7	27.8 ± 4.9	26.3 ± 4.0	25.8 ± 2.6	< 0.001
**Diet quality score (overall healthy dietary pattern)**	4.46 ± 0.81	4.82 ± 1.16	5.51 ± 1.26	6.51 ± 0.72	< 0.001
**Overall healthy dietary pattern components**					
Fruit (serving/day)	1.91 (1.18 – 3.29)	2.33 (1.38 – 3.98)	3.11 (1.73 – 5.07)	4.49 (3.17 – 6.44)	< 0.001
Vegetables (serving/day)	2.47 (1.71 – 3.52)	2.53 (1.69 – 3.79)	3.19 (2.04 – 4.38)	3.66 (2.85 – 4.88)	< 0.001
Dairy products (serving/day)	1.42 (0.86 – 2.08)	1.56 (1.05 – 2.23)	1.70 (1.11 – 2.52)	2.28 (1.45 – 3.09)	< 0.001
Whole grains (serving/day)	2.77 (1.42 – 5.05)	2.90 (1.56 – 5.17)	3.84 (2.06 – 6.28)	4.41 (3.03 – 7.30)	< 0.001
Fish (serving/day)	0.21 (0.11 – 0.42)	0.21 (0.11 – 0.43)	0.23 (0.12 – 0.48)	0.29 (0.14 – 0.54)	< 0.001
Nuts (serving/day)	0.13 (0.06 – 0.24)	0.14 (0.07 – 0.30)	0.16 (0.07 – 0.33)	0.21 (0.10 – 0.43)	0.001
Red meat (serving/day)	0.66 (0.40 – 0.96)	0.64 (0.38 – 1.02)	0.63 (0.37 – 1.02)	0.61 (0.37 – 1.03)	0.144
Processed meat (serving/day)	0.08 (0.02 – 0.15)	0.08 (0.02 – 0.15)	0.07 (0.01 – 0.17)	0.07 (0.01 – 0.16)	0.607
Refined grains (serving/day)	4.02 (2.97 – 6.10)	4.17 (2.89 – 6.54)	3.88 (2.80 – 5.98)	3.65 (2.37 – 5.48)	0.001
Sweetened beverage (serving/week)	1.12 (0.42 – 2.80)	0.91 (0.35 – 2.10)	0.77 (0.28 – 1.96)	0.70 (0.21 1.82)	< 0.001
Sodium (mg/day)	3655 (3002 – 4748)	3445 (2719 – 4302)	3287 (2558 – 4155)	3336 (2659 – 4118)	< 0.001
Trans fatty acid (g/day)	2.35 (1.14 – 4.72)	2.21 (1.19 – 4.58)	2.39 (1.25 – 4.71)	2.35 (1.21 – 4.51)	0.651

Data are presented as mean ± standard deviation for normally distributed variables and median (25–75 interquartile range) for skewed variables, and percent for categorical variables

^a^Percentage of diabetes incidence across quartiles of HLS- HDP was determined at the end of follow-up of study

According to Table 3, individuals in the highest quartile of the HLS-mPNNS-GS score significantly to be more female, younger, high-active, more employed, lower-smoked, had higher level of academic education, and had lower BMI, TGs, FPG, LDL-C, SBP, and DBP levels (at baseline) and lower % incidence of T2D (after 6-years of follow-up) compared to those in the lowest quartile of this score (*P* < 0.001). Also, based on our results, the intakes of vegetables and fruits, bread and legumes, whole grains, seafood, and vegetable oils significantly increased across quartile of the HLS-mPNNS-GS score, whereas the intake of red meat significantly decreased across quartile of the HLS-mPNNS-GS score (*P* < 0.05), (Table 3).

According to Table 4, participants in the highest quartile of the HLS-HDP score significantly to be more female, younger, non-smoked, more employed, had higher levels of academic education, physical activity, and HDL-C, and had lower levels of BMI, FPG, TGs, SBP, DBP (at baseline) and lower % incidence of T2D (after 6-years of follow-up) compared to those in the lowest quartile (*P* < 0.05). Also, our findings showed that the intakes of vegetables, fruits, dairy products, nuts, whole grains, and fish significantly increased across quartiles of HLS- HDP score (*P* < 0.05), whereas dietary intakes of refined grains, sodium, and sweetened beverage were significantly reduced across quartiles of HLS- HDP score (*P* < 0.05).

**Fig. 2:**
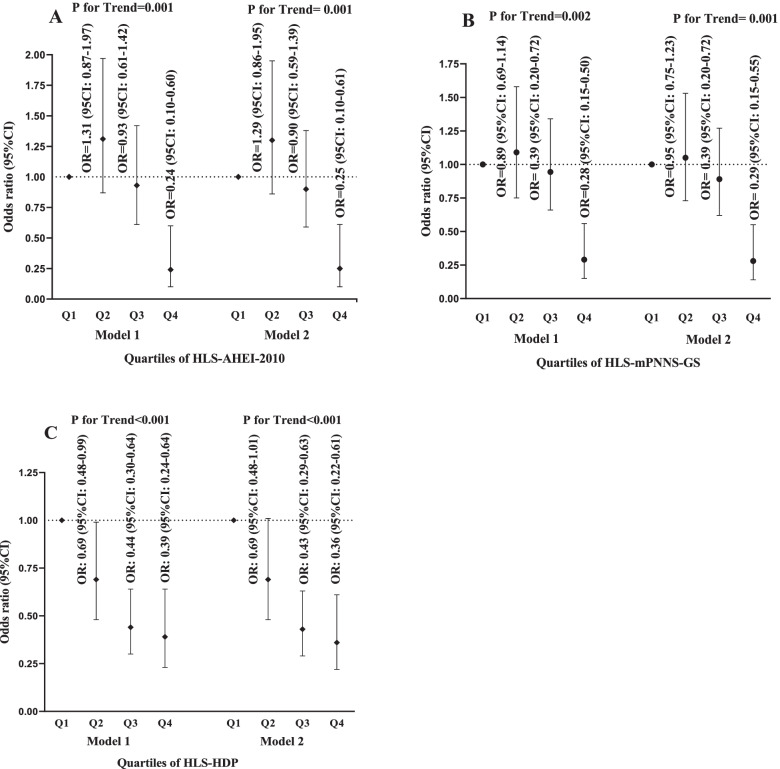
**A-C **The odds ratio (OR) and 95% confidence interval (CI) of type 2 diabetes across quartiles of healthy lifestyle scores [HLS-AHEI-2010 (**A**), HLS-mPNNS-GS (**B**), and HLS-HDP (**C**)] based on model 1(adjusted for age and sex) and model 2 (adjusted for sex, age, daily energy intake, occupational status, educational level, and marital status)

We determined the odds of T2D across the quartiles of HLS-AHEI-2010, HLS-mPNNS-GS, and HLS-HDP and showed main results in Fig. 2 (A-C). Based on the age and sex-adjusted model, the odds of T2D was reduced across quartiles of HLS-AHEI-2010 (OR = 0.24; 95%CI: 0.10–0.60, *P* for trend = 0.001), HLS-mPNNS-GS (OR = 0.28; 95%CI: 0.15–0.50, *P* for trend = 0.002), and HLS-HDP (OR = 0.39; 95%CI: 0.24–0.64, *P* for trend < 0.001). Also, findings of the multivariable-adjusted model indicated that after adjusting age, gender, energy intake, employment status, education, and marital status, the risk of T2D was decreased across the quartiles of HLS-AHEI-2010 (OR = 0.25; 95%CI:0.10–0.61, P for trend:0.001), HLS-mPNNS-GS (OR = 0.29; 95%CI: 0.15–0.55, P for trend:0.001), and HLS-HDP (OR = 0.36; 95%CI: 0.22–0.61, *P* for trend < 0.001).

## Discussion

The current study has provided notable results on the association of HLS and the risk of T2D in the framework of longitudinal population-based study after a 6-year follow-up. Our findings reported that a higher score of HLS, assessed by three indices including HLS-AHEI-2010, HLS-mPNNS-GS, HLS-HDP, is related to decreased risk of T2D independent of confounding factors.

Although strong reviews have focused on the association of each lifestyle factor with the risk of T2D incident separately, their findings have shown a significant association of healthy dietary pattern scores such as DASH diet, Mediterranean diet, and healthy eating index [12], vigorous physical activity [10], smoking [9], and elevated BMI with risk of T2D [11], there is no study on the combined role of lifestyle factors score as a single variable with risk of T2D. Recently, some studies have investigated the combined role of HLS in health status or chronic diseases risk, such as the risk of coronary disease [14], healthy aging [13], and mortality [15, 16]. Interestingly, our study results are in agreement with the results of two previous studies that observed an inverse association between the HLS and total mortality in adults with type 2 diabetes patients [15, 16]. Also, in the Atallah et al. study, a one-point increase in the HLS was associated with an 11% higher probability of healthy aging [13]. Furthermore, the Khera et al. study has reported that in individuals with high genetic susceptibility, a healthy lifestyle was associated with a 46% lower risk of coronary disease events than an unfavorable lifestyle [14].

For investigation of the relationship between HLS and T2D risk, we have determined HLS in participants using three indices, including HLS-AHEI-2010, HLS-mPNNS-GS, and HLS-HDP. The rational for using these three indices was as follows: first, the scoring procedure of these indices were different in diet quality assessment or considering the cut point for the classification of participants based on BMI level; e.g. Patel et al. had used the AHEI 2010 to assess the diet quality [15], however, the Atallah et al. had used the PNNS-GS method to determine food quality [13]. Also, in Patel et al. and Atallah et al. studies, subjects were divided into two groups of individuals with normal weight and those with overweight and obesity, but in Khera et al. study, individuals with normal weight and overweight were classified into one group and received the same score [14]. Second, each of these HLS scoring methods has been used and validated in different societies that their population probably was different in general characteristics such as lifestyle and dietary pattern with other population such as people in Middle East and North Africa (MENA) region. Another important point was that in the previous studies, each of these indices has been used to determin the HLS in relation to mortlity or various nutrition-related chronic diseases risk, but none of previous studies has been focused on risk of the T2D. Therefore, in our study, we assessed the relationship of healthy lifestyle, determined by three HLS indices, with the T2D risk in the Iranian population. Based on the results of all three methods, we were able to show a remarkable association between a higher HLS score and reduced the T2D risk, which indicates that all three methods used have an acceptable and similar ability in the prediction of people's healthy lifestyle score and its possible relationship with the incidence of T2D.

The results of the current study are further in agreement with the results of previous studies that have summarized evidence on the independent role of individual lifestyle factors in the development of T2D [9–12]; one of the most important components of a lifestyle affecting the incidence of T2D is the dietary pattern of individuals. A systematic review has shown that the higher adherence to healthy dietary patterns such as the Mediterranean diet and the DASH diet, characterized by higher consumption of fruits, vegetables, legumes, nuts, low-fat dairy, whole grain, seafood, dietary fiber, unsaturated fats, and antioxidant nutrients and lower consumption of animal fat, red and processed meat, sweets and desserts, are associated with decreased the risk of T2D [12]. Also, the greater adherence to alternative healthy eating index focused on a high intake of vegetables, fruits, nuts, legumes, whole grain, PUFA, and lower intakes of red meat, sweet beverages, trans fatty acids, and sodium, was linked to decreased the risk of T2D as well as lower prevalence of general obesity [12].

Several studies have also focused on the independent role of other lifestyle factors, including smoking, physical activity, and obesity, in the pathogenesis of T2D. In a meta-analysis of cohort studies, higher physical activity was related to a substantially lower risk of T2D [10]. It has been suggested that the vigorous physical activity level has a protective role in reducing the risk of T2D via the beneficial effect on insulin sensitivity, improving glucose metabolism via insulin- receptor up-regulation in muscle, and increased insulin and glucose delivery to muscle, increasing total antioxidant capacity and reducing central adiposity through negative energy balance [24, 25]. Also, the Akter et al. study has reported that cigarette smoking is linked with an increased risk of T2D [9]. Smoking leads to inadequate insulin secretion and insulin resistance [26, 27] via various underlying effects such as hormonal imbalance, inflammation, oxidative stress, central adiposity, and endothelial dysfunction [9, 28]. Also, smoking harms the metabolism of nutrients, β-cells dysfunction, and up-regulating inflammatory markers such as C-reactive protein (CRP), which can be related to increased risk of cardiometabolic abnormalities [29, 30]. Furthermore, the elevated body weight and central adiposity is another main underlying risk factor for T2D [11]; because obesity has an important role in various metabolic pathways and in producing several potential risk factors such as increased levels of the chronic inflammatory response (increased inflammatory markers such as interleukin-6 and tumor necrosis factor-alpha), increased risk of impaired hepatic free fatty acids and glucose metabolism, hyperinsulinemia, β-cell dysfunction, insulin resistance, and dyslipidaemia which all of them are mostly associated with higher risk of T2D [31, 32].

Given the important individual role of each lifestyle factor in predicting the risk of T2D, we examined the relationship between the combined role of these factors and the risk of T2D in individuals who followed a healthy lifestyle. Our results provide strong evidence that following combined healthy behaviors in the framework of a healthy lifestyle, which are characterized by a healthy dietary pattern rich in fruits and vegetables, legumes and grains, lower consumption of red and processed meat and sweetened beverage, and having regular meals, high physical activity, and abstinence from smoking, can have a remarkable effect size in reducing the risk of T2D incident. Indeed, despite the differences between the previous studies and the current study concerning definitions of HLS (components and scoring), the robust findings reinforced and confirmed the assumption that combined healthy behaviors may positively impact health promotion and decrease risk of chronic diseases such as T2D.

Our study has several important strengths. This study is the first population based prospective study that assessed the combined role of healthy lifestyle factors in prediction of T2D risk. Also, the current study has a large sample size and relatively long-term follow-up duration. Using valid and reliable food-frequency and physical activity questionnaires for collection the participants’ data on dietary intakes and physical activity levels is another strength of this study. Despite these strengths, some limitations of current study should be mentioned. Similar to epidemiological studies, recall bias and measurement error are inherent limitation of using the FFQ in the present study. Also, although various confounding factors were controlled in multivariable logistic regression model, we cannot ruled out the effects of residual or unmeasured confounders.

## Conclusion

In conclusion, our findings have shown that higher adherence to HLS, characterized by a healthy diet, vigorous physical activity, not smoking, and normal BMI, is strongly associated with a decreased risk of T2D in Iranian adults. Further observational studies are suggested to assess the combined protective role of HLS in the development of T2D among other populations.

## Data Availability

The datasets analysed in the current study are available from the corresponding author on reasonable request.

## Abbreviations

AHEI-2010: Alternate healthy eating index-2010
AHA: American Heart Association
BMI: Body mass index
CI: Confidence interval
CVD: Cardiovascular disease
DBP: Diastolic blood pressure
FPG: Fasting plasma glucose
FCT: Food composition table
FFQ: Food frequency questionnaire
HDL-C: High-density lipoprotein cholesterol
HDP: Healthy diet pattern
HLS: Healthy lifestyle score
LDL-C: Low-density lipoprotein-cholesterol
MAQ: Modifiable Activity Questionnaire
MET-h/wk: Metabolic equivalent hours per week
mPNNS-GS: Modified French Programme National Nutrition Santé-Guideline Score
SBP: Systolic blood pressure
TC: Total cholesterol
TG: Triglyceride
TLGS: Tehran Lipid and Glucose Study
T2D: Type 2 diabetes
USDA: United States Department of Agriculture
WC: Waist circumference
